# Supplementary material for: Long-Term Functional Rescue of Trauma-Induced Vision Loss by a Novel, Small Molecule TrkB Activator
Source: bioRxiv. 2025 Feb 23:2025.02.18.638863. Preprint. [Version 1] doi: 10.1101/2025.02.18.638863 (PMC11870575; doi:10.1101/2025.02.18.638863)
Supplement: 1 [file NIHPP2025.02.18.638863V1-supplement-1.pdf]

bioRxiv preprint doi: https://doi.org/10.1101/2025.02.18.638863; this version posted February 23, 2025. The copyright holder for this preprint (which was not certified by peer review) is the author/funder, who has granted bioRxiv a license to display the preprint in perpetuity. It is made available under aCC-BY 4.0 International license.

Supporting Information

Figure S1-1. Chemical synthesis of HIOC (**1**) and analogs **2** - **6**. Differences in analog structures from HIOC are highlighted in red.

Figure S2. Time course of protium-deuterium exchange with HIOC (**1**) at methine carbon, at pD 7.8

Figure S3: Thermal ellipsoid representation of the asymmetric unit of HIOC (**1**). There is one molecule of the target compound in the asymmetric unit, which is represented by the reported sum formula. In other words: Z is 2 and Z' is 1. The chiral centers have both R and S-configuration in this crystal.

Figure S4: Plot of a portion of the hydrogen bonding network in the crystal structure of HIOC (**1**).

Figure S5: The molecular structure of HIFN (**5**).

Figure S6: Hydrogen bonded dimer in the crystal structure of HIFN (**5**).

Figure S7. Analog 6 did not exhibit in-vivo neuroprotection. Animals were treated with analog **6** (ip, 40mg/kg) following blast (same regime was followed as for HIFN (**5**) treatment shown in Figure 2A). No statistically significant change in P1 (A) and N2 (B) wave was seen in animals treated with analog **6** compared to vehicle treated animals. Analog **6** treatment did not rescue the visual function deficit; no statistically significant improvement in contrast sensitivity (C) and visual acuity (D) was observed in treated animals. * $p \leq 0.05$; ** $p \leq 0.01$;**** $p \leq 0.0001$, n=4-5/group.

Figure S8. Outer retinal neurons were not affected by overpressure blast injury. At 9 weeks post blast, animals did not show any change in rod photoreceptor or rod bipolar cell function as assessed by ERG: (A) amplitude of 'a-wave'; (B) amplitude of 'b-wave'. Function of RPE and amacrine cells was also not altered as shown by: (C) 'c-wave' and (D) oscillatory potentials (OP), respectively**.** No significant differences were found for any of the parameters recorded. Data are expressed as mean ± SEM; n=6-8/group.

Figure S9. HIFN reduces visual function decline in females and males. Neuroprotection by HIFN was tested in female mice. Mice were exposed to blast and treated with HIFN (40 mg/kg) or vehicle 30 minutes later and daily for the next 6 days. Visual acuity was measured 8 days after blast exposure. ** $p \leq 0.01$; *** $p \leq 0.001$; **** $p \leq 0.0001$, n=5-6/group.

Table S1. Tabulated ^{13}C and ^{1}H NMR data for HIFN (**5**)

Table S2. Electroretinogram 'a-wave' and 'b-wave' recordings 3 and 6-week post blast showing no significant effect on rod photoreceptor and bipolar cell function.
